# Evaluation of a Method to Determine Wear Resistance of Class I Tooth Restorations during Cyclic Loading

**DOI:** 10.3390/ma15155440

**Published:** 2022-08-08

**Authors:** Philipp Messer-Hannemann, Mariam Samadi, Henrik Böttcher, Sebastian Duy, Daniela Duy, Niclas Albrecht, Falk Schwendicke, Susanne Effenberger

**Affiliations:** 1 DMG Dental-Material Gesellschaft mbH, 22547 Hamburg, Germany; 2 SD Mechatronik GmbH, 83620 Feldkirchen-Westerham, Germany; 3 Department of Oral Diagnostics, Digital Health and Health Services Research, Charité-Universitätsmedizin Berlin, 14197 Berlin, Germany

**Keywords:** glass ionomer, dental restorative material, chewing simulation, Class I restoration, wear resistance

## Abstract

The aim of this study was the development of a test regime to determine the wear resistance and predict the clinical performance of conventional glass ionomer cement (GIC) restorations in Class I tooth cavities. Cavities were prepared in excised human teeth and restored using three conventional glass ionomer restorative materials: DeltaFil, Fuji IX GP and Ketac Universal. The restored teeth were mechanically and thermally stressed using a chewing simulator with a maximum number of 1,200,000 load cycles. Besides determining the number of cycles achieved, the abrasion volume after termination of the chewing simulation was calculated using µCT images. All teeth restored with DeltaFil reached 1,200,000 cycles without any restoration failure. Only 37.5% of the restorations each with Ketac Universal and Fuji IX GP were able to achieve the maximum cycle number. A significant lower abrasion volume for restorations with DeltaFil compared to Ketac Universal (*p* = 0.0099) and Fuji IX GP (*p* = 0.0005) was found. Laboratory chewing simulations are a useful tool to study basic wear mechanisms in a controlled setting with in-vivo related parameters. DeltaFil shows an improved wear resistance compared to other conventional GICs, indicating the high potential of this material for long-lasting Class I restorations.

## 1. Introduction

The aim of direct restorative materials in dentistry is to apply a biocompatible and durable material that can permanently bind to the tooth structure and has a reasonable high wear resistance to withstand mechanical loads during mastication and bruxism. Besides resin-based composites as a permanent solution for occlusally stressed areas or areas with high aesthetic demands, glass ionomer cement (GIC) is a traditional and well-established alternative for restoring dental hard tissue. GICs are particularly used in pediatric dentistry with a generally high caries risk and for patients who need more demanding treatments such as elderlies or patients with comorbidities [1,2]. According to the World Health Organization (WHO), GIC was recently included in the list of essential medicines to cover minimum medicine needs for a basic health-care system [3].

Conventional GICs are created by an acid-base reaction between a liquid that mainly consists of an aqueous solution of a polyacid and a powder that contains fluoro-alumino-silicate glass fillers [4]. Due to the chemical composition of conventional GICs, washing out of unpolymerized monomers or components of the initiator system, which can have harmful effects on health, can be avoided compared to resin-based materials [5]. This and the ability to chemically bond with the tooth surface underline the excellent biocompatibility of direct restorative dental materials based on GICs [6,7]. Conventional GICs are therefore independent of a light curing unit or an additional bonding agent, which makes the treatment technically less sensitive compared to resin-based composites [8], thus reducing the risk of marginal leakage at the interface between restoration and tooth and enabling atraumatic restorative treatment (ART) for patients with low compliance [9,10]. Another benefit of using a GIC as restorative material is the release of fluoride ions to reduce the risk of secondary tooth decay and to support the remineralization process of dental hard tissue [11,12,13].

The main drawback of conventional GICs is their limited mechanical properties, i.e., flexural strength, wear resistance at sites exposed to higher occlusal forces and fracture toughness. This limits their use as a permanent direct restorative material in stress-bearing areas [14,15]. Resin-modified GICs are less susceptible to the formation of cracks due to their superior mechanical properties [16,17]. However, the polymerized resin matrix limits ion exchange with the oral environment leading to a decreased fluoride ion release rate, which reduces the ability of resin-modified materials to prevent caries progression [18,19]. The addition of monomers, which enable a polymerization reaction of the material initiated by light activation, also limits the acid-base reaction and reduces biocompatibility compared to conventional GICs [20]. Numerous attempts have been made in the past to improve the properties of conventional GICs [21,22]. For example, they have been reinforced with inorganic nanoparticles such as alumina or hydroxyapatite crystals to improve their mechanical properties and to avoid the downsides of adding a resin, which would compromise the benefits of a biocompatible restorative material [23,24,25]. In this study, DeltaFil (DMG, Hamburg, Germany), an innovative conventional GIC with dispersed polymeric micelles was used to prevent crack propagation, thus aiming to improve the fracture toughness compared to well-established conventional GICs. The concept of dispersing ductile particles to improve the fracture resistance of a brittle material is a well-known concept in materials science [26,27]. Transferring this concept to dentistry to reinforce glass ionomer restorative materials is a new approach to extend the survival of conventional glass ionomer restorations.

Direct restorative materials are subjected to complex wear mechanisms in the human oral environment including abrasion, attrition, adhesion and corrosive wear or any combination of these mechanisms [28,29]. Determination of the wear resistance in-vitro is a challenging task, and no system of chewing simulation is able to reproduce the dynamic movements of human mastication under the actual humidity and pH conditions in the oral environment [30]. However, several test methods are reported to evaluate the wear of dental restorations i.e., bridges and crowns, showing that using a chewing simulator is a good indicator to predict the clinical outcome to some extent [31,32]. So far, most studies regarding the wear resistance of direct restorative materials were performed without the application of the material to restore a cavitated tooth [31,33,34]. Since there is a lack of well-validated laboratory methods for predicting the clinical performance of direct restorative materials, the aim of this study was to transfer the in-vitro test procedure evaluating the wear performance of prosthetic materials to glass ionomer restorations. In this way, the closest possible approximation to clinical studies can be achieved, which yet will always be necessary for the final assessment of clinical suitability. Nevertheless, it should be noted that material parameters, determined in standardized and validated laboratory tests, can only be correlated with clinical data to a certain extent, since clinical assessment of material related parameters offers a variety of possible reasons for variability, such as factors related to the patient, the skill of the dentist or the treatment itself [35]. The aim of this study was rather to develop a standardized in-vitro test protocol in order to determine the suitability of conventional GIC as durable direct restorative material in Class I cavities. As part of this research question, the study further investigated to what extent the reinforcement of conventional GICs can improve the fracture toughness of the material and the durability of the restoration.

## 2. Materials and Methods

Three conventional glass ionomer restorative materials have been analyzed (Table 1): DeltaFil (DMG, Hamburg, Germany), Fuji IX GP (GC, Tokyo, Japan) and Ketac Universal (3M, St. Paul, MN, USA). For investigating differences between the restorative materials, a suitable in-vitro test procedure for determining the wear resistance of direct tooth restorations had to be developed, as existing procedures were only available for the mechanical loading of dental crowns and bridges, which are not necessarily suitable to evaluate the wear resistance of direct restorative materials. The fracture toughness of the materials was assessed in a notchless triangular prism (NTP) test.

### 2.1. Wear Resistance

Class I cavities (2 × 2 × 2 mm^3^) were prepared in 24 extracted sound human teeth (mandibular first molars) and restored using the investigated restorative materials (*n* = 8/group). The GICs were applied according to the instructions for use with a pre-defined liquid-powder mixing ratio using capsules provided by the manufacturer. The capsules were activated according to the respective specifications and mixed in a capsule mixer (Silamat S6, Ivoclar Vivadent). The mixed glass ionomer restorative material was then applied to the prepared cavities using the manufacturer’s corresponding dispensers. For DeltaFil and Fuji IX GP, an additional step of pre-treatment of the cavity was carried out using a conditioner provided by the respective manufacturer, while Ketac Universal was applied directly into the cavity. After application of the material, excess was removed and the restorations were modelled and polished once the material was cured. While the restorations made with DeltaFil and Ketac Universal were neither coated nor varnished according to the instructions for use, the restorations made with Fuji IX GP were coated with Fuji VARNISH (GC, Tokyo, Japan) in an additional step. No color matching of the filling material to the tooth color was made. The restored teeth were then stored in distilled water at 37 °C for 24 h.

**Figure 1:**
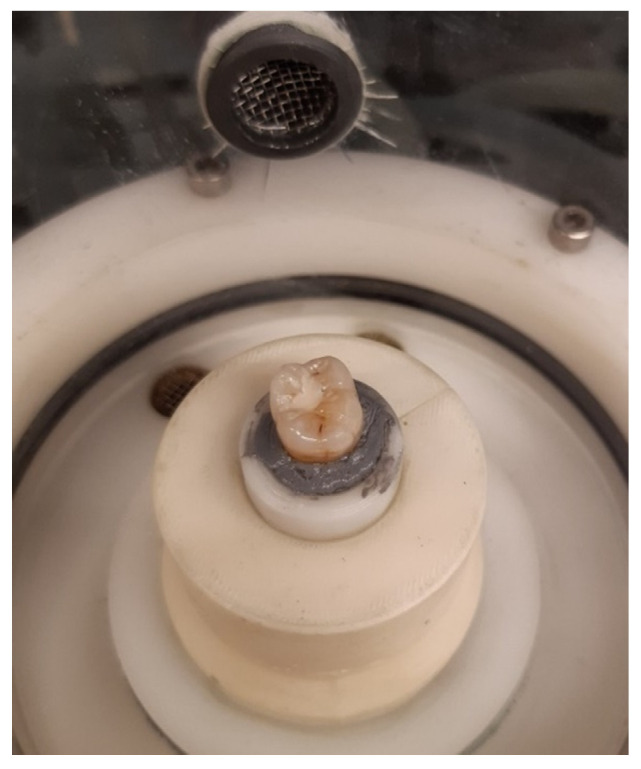
Chewing simulation setup with a restored tooth (DeltaFil—Specimen 4) mounted inside the chamber of the chewing simulator.

Until the start of the test, the samples were stored in a humid environment. Before mounting the specimens into the chambers of the chewing simulator (CS-4.8, SD Mechatronik GmbH, Feldkirchen-Westerham, Germany), the restored teeth were glued into specific holders using silicone putty and a two-component adhesive (Figure 1). A steatite cone with tip radius of 1.5 mm and 30° cone angle was used as antagonist to transfer the load to the restored tooth. Steatite is well established as an antagonist in chewing simulations [36,37,38]. As such, it is considered to be a suitable substitute for enamel in order to act as a standardized antagonist allowing quantitative assessment of the wear behavior [39,40]. The antagonist was visually positioned above the fissure of the tooth and brought into contact with the specimen to define the starting point of the chewing simulation.

The specimens were subjected to a vertical load of 50 N and underwent a combined vertical and lateral cyclic movement (f = 1.4 Hz) with a displacement amplitude for the vertical movement of 2 mm and for the lateral movement of 1.5 mm. Simultaneously, the mounted teeth were thermally stressed (5 °C/55 °C, 60 s each cycle) using distilled water as transport medium in order to mimic the environment in the oral cavity during food consumption [36,41]. For this purpose, flooding, and evacuation of the chamber with water of different temperatures was integrated into the testing rig. Failure of the specimen or a maximum number of 1,200,000 load cycles was defined as the termination criterion for the chewing simulation [42]. Once the maximum number of load cycles was reached, a total of approximately 6500 thermal cycles had been run through accordingly. Failure of the restored tooth was defined when the maximum abrasion depth of 2 mm was reached. Loading of the specimen created a gap between the crosshead and the weight support of the chewing simulator, which corresponds to the downward stroke of the vertical displacement. As soon as this gap had completely disappeared, the maximum abrasion depth was reached. This visual check was done at regular time intervals during the chewing simulation.

**Figure 2:**
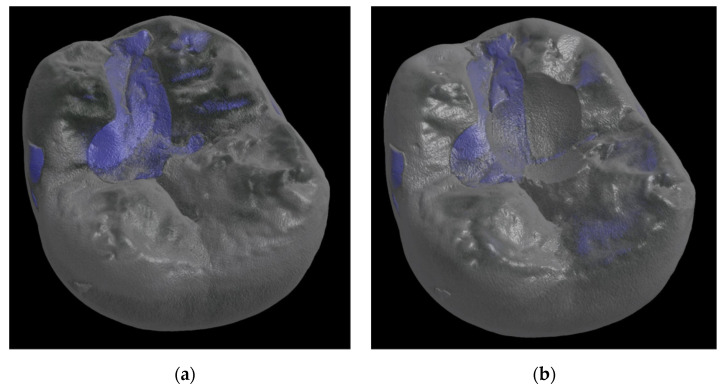
Reconstructed µCT images of a restored tooth to calculate the abrasion volume (DeltaFil—Specimen 8). The µCT images were recorded prior to the chewing simulation (**a**) and after completion of the chewing simulation at 1,200,000 cycles (**b**).

In addition to determining the number of cycles achieved, the abrasion volume after termination of the chewing simulation was calculated using µCT images (Skyscan 1275, Bruker AXS, Karlsruhe, Germany). µCT images (80 kV, 125 µA) of the restorations were recorded prior to cyclic testing and after removal of the tooth from the testing rig to determine the abrasion volume as volumetric difference between the reconstructed 3D images using the 3D visualization software Avizo (Thermo Fisher Scientific, Waltham, MA, USA). To calculate the volumetric difference, pre- and post-scans of the teeth were superimposed as volume models based on their geometric features (Figure 2). In addition to the abrasion volume, detection of possible flaws, inclusions, or voids in the abrasion surface of the specimens could be enabled with the µCT images.

### 2.2. Fracture Toughness

The fracture toughness of the glass ionomer materials was measured in a separate experiment using 6 mm × 6 mm × 6 mm × 12 mm notchless triangular prism (NTP) test specimens (*n* > 14), accordingly to the test developed and described by Ruse et al. [43]. After application of the restorative materials into an appropriate test specimen mold with the required dimensions, the cured specimens were ground to an edge length of 6 mm using a SiC abrasive paper (P2000) in a wet grinding process. The test specimens were conditioned in distilled water at 37 °C for 24 h before being mounted in the loading frame. A small defect with a maximum depth of 0.1 mm was introduced into the specimens using a gauge to initiate targeted crack propagation. Loading of each specimen was done with a speed of 1.0 mm/min until fracture occurred using a micro-tensile-device (MTD-500, SD Mechatronik GmbH, Feldkirchen-Westerham, Germany). According to the instructions for use, the Fuji IX GP specimens were coated with Fuji VARNISH (GC, Tokyo, Japan) in an additional step, whereas no coating or varnish was applied to the DeltaFil and Ketac Universal specimens. The maximum load *F_max_* at fracture of the specimen was used to calculate the fracture toughness *K_IC_* using the following equation:

(1) $$K_{IC}=Y^{*}{}_{min}\frac{F_{max}}{D\sqrt{W}}$$

with Y*_min_ = 28, *D* = 12 mm and *W* = 10.4 mm [43].

### 2.3. Statistical Analysis

Statistical analysis was performed using GraphPad PRISM (Graphpad Software Inc., San Diego, CA, USA). The distribution of the data was tested with the Kolmogorov–Smirnov test and according to the test results, parametric or non-parametric approaches were used to analyze the data. The results of the chewing simulation (number of cycles, abrasion volume) were analyzed for statistically significant differences using the non-parametric Kruskal–Wallis test and pairwise comparisons (Dunn’s test). The fracture toughness of the materials investigated was compared using one-way analysis of variance (ANOVA) and Tukey post-hoc test. A Type I error level of 0.05 was used for all tests of significance.

## 3. Results

### 3.1. Wear Resistance

All teeth restored with DeltaFil reached the maximum number of 1,200,000 cycles without any restoration failure. In comparison, only three out of eight restorations each with Ketac Universal and Fuji IX GP achieved the maximum number of cycles (Table 2).

**Figure 3:**
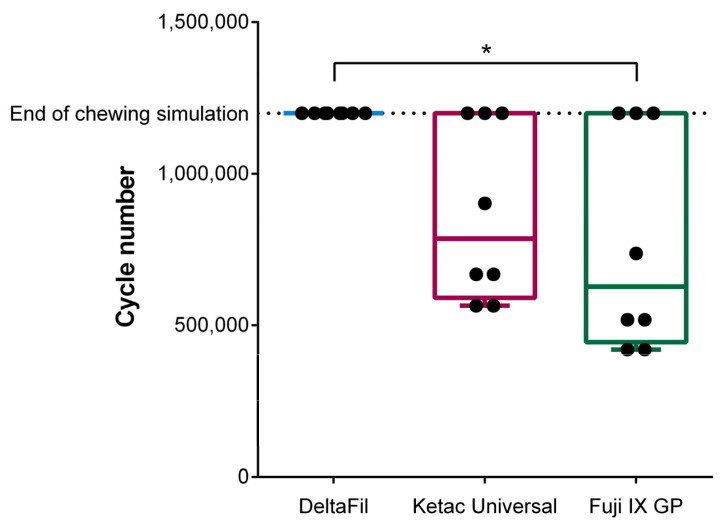
Cycle number of the restored teeth at termination of the chewing simulation. All teeth restored with DeltaFil reached the maximum number of 1,200,000 cycles. Restorations with DeltaFil lasted significantly longer than restorations with Fuji IX GP (‘*’: *p* ≤ 0.05). The error bars represent the minimum and maximum values of the measured data.

Whereas no significant difference between the reached cycle number for restorations with Ketac Universal and Fuji IX GP was found (*p* > 0.9999), restorations with DeltaFil lasted significantly longer than restorations with Fuji IX GP (*p* = 0.0204) (Figure 3). Although the cycle number of DeltaFil clearly exceeds that of Ketac Universal, no significant difference was found between the two materials (*p* = 0.1255).

**Figure 4:**
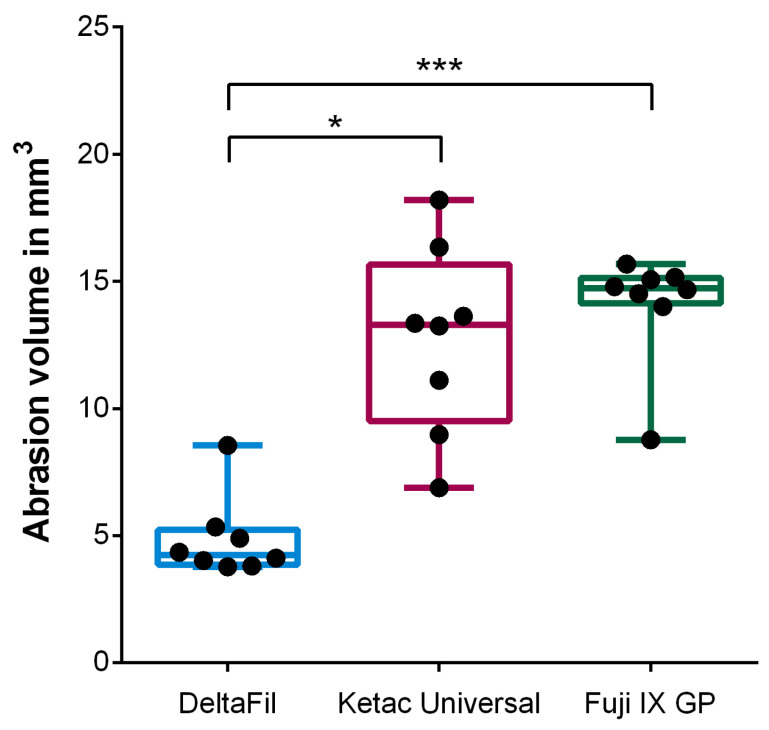
Abrasion volume of the restored teeth after termination of the chewing simulation. Restorations with DeltaFil resulted in a significantly decreased abrasion volume compared to Ketac Universal and Fuji IX GP (‘*’: *p* ≤ 0.05; ‘***’: *p* ≤ 0.001). The error bars represent the minimum and maximum values of the measured data.

Evaluation of the reconstructed 3D µCT images prior to testing and after termination of the chewing simulation resulted in a significant lower abrasion volume for restorations with DeltaFil compared to Ketac Universal (*p* = 0.0100) and Fuji IX GP (*p* = 0.0005) (Figure 4). No significant difference was found between Ketac Universal und Fuji IX GP regarding the abrasion volume (*p* > 0.9999).

**Figure 5:**
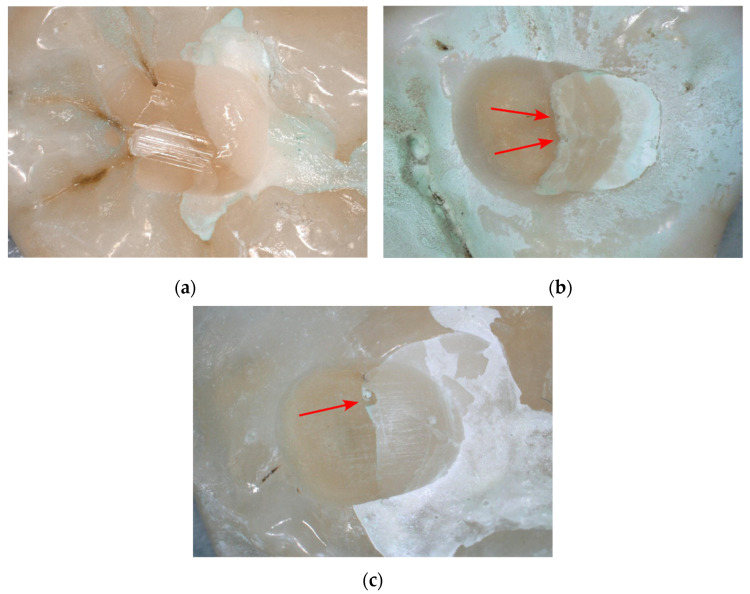
High resolution photographs showing the abraded surfaces of the restorations at termination of the chewing simulation. (**a**) DeltaFil (specimen 5; 1,200,000 cycles), (**b**) Ketac Universal (specimen 6; 668,629 cycles), (**c**) Fuji IX GP (specimen 5; 419,892 cycles). Red arrows indicate chipping and embrittlement of the restoration at the dentin interface.

After termination of the chewing simulation at the maximum number of 1,200,000 load cycles, the interface between DeltaFil and dentin appeared very smooth without any indications of chipping or delamination of the restoration (Figure 5a). For restorations with Ketac Universal and Fuji IX GP that did not reach the maximum cycle number, chipping of the restorative material and signs of embrittlement could be observed at the interface to dentin (Figure 5b,c).

### 3.2. Fracture Toughness

**Figure 6:**
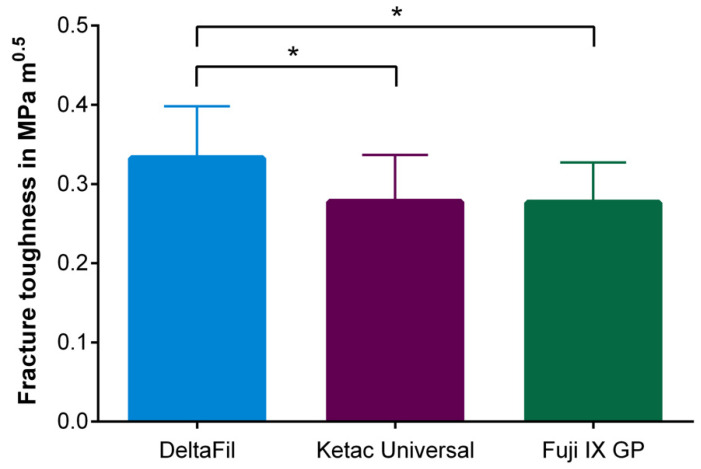
Fracture toughness of the investigated GICs measured using a notchless triangular prism testing rig. The fracture toughness of DeltaFil was significantly higher compared to Ketac Universal and Fuji IX GP (‘*’: *p* ≤ 0.05). The error bars represent the standard deviations of the measured data.

During the sample preparation for measuring the fracture toughness of the materials investigated, four specimens had to be excluded from the statistical analysis due to insufficient dimensions and three specimens due to geometric defects. Three specimens were marked as outliers after the fracture toughness test was carried out and were thus also excluded from the analysis. The one-way ANOVA revealed that there was a significant difference between the restorative materials used (*p* = 0.0150, η^2^ = 0.1812). Tukey post hoc test for multiple comparisons found that the mean fracture toughness of DeltaFil was significantly higher compared to Ketac Universal (*p* = 0.0468) and Fuji IX GP (*p* = 0.0235), whereas no significant difference was observed between Ketac Universal and Fuji IX GP (*p* = 0.9988) (Table 3, Figure 6).

## 4. Discussion

Since the use of amalgam as cost-effective, mechanically stable direct restorative material must be phased down due to regulations regarding mercury pollution [44], there is an increased need for substitute materials to fill this gap. As consequence, validated in-vitro studies are becoming increasingly important to predict the clinical performance of direct restorative materials in advance [37,42]. However, clinical correlation of wear between in-vivo data and results from chewing simulation tests for direct restorative materials are rare. Most studies that include chewing simulation tests concentrated on the fatigue resistance of crowns and bridges [36,38]. In this study, a chewing simulator was used to simulate two-body wear representative of the contact between a cavitated tooth restored with a conventional glass ionomer restorative material and an antagonist during mastication or bruxism.

Only the Class I restorations made with DeltaFil reached the maximum number of 1,200,000 cycles without any early failure of one of the specimens. A load of 50 N in combination with a maximum number of 1,200,000 load cycles was chosen, as these parameters are well-established in the investigation of crowns and bridges to imitate five years of intraoral function within a chewing simulation [42,45]. Since conventional glass ionomer restorative materials are generally limited to permanently withstand high occlusal loads, the relatively low load of 50 N was deemed appropriate. However, it is very difficult to correlate the wear behavior of the GICs used in this study with in-vivo data or even other chewing simulator tests, as the methodology of the chewing simulation testing rigs is very different. This includes the type of the opposing material, the magnitude of loading force and movement patterns as critical factors when wear tests are designed to simulate the conditions in the oral environment. Since wear is not an intrinsic property of a specific material, there is a lack of comparable methods to determine the wear resistance of restorative dental materials [29]. The presented chewing simulation test involved a steatite cone as antagonist, which transferred the acting force directly to the restoration. Although using steatite as antagonist might lack clinical relevance in comparison to enamel or an antagonist tooth to transfer the load, chewing simulation tests of restorations, which were opposed by standardized steatite antagonists, allow for a quantitative evaluation of the results and a comparison of the glass ionomer restorative materials investigated [40]. Since the surface area of the prepared Class I cavities was rather small, a cone-shaped antagonist was ideal to be positioned well in the fissure. Another limiting factor of this experimental set-up is the use of water instead of artificial saliva as a transport medium for thermocycling. Artificial saliva alters the lubrication regime between the antagonist and the glass ionomer restorative material, which in turn may affect the wear rate of the restoration [46,47]. The wear rate can also be accelerated by acidic substances in interaction with high occlusal loads [48]. Further investigations should also include adhesive wear, third-body wear, and corrosive wear to cover the full range of wear mechanisms that can occur in the oral environment. The relatively small sample size was selected according to the capacity of the chewing simulator that was used testing all restored teeth at the same time. With this we were able to ensure that all teeth were treated equally during restoration, storage and testing. Furthermore, the primary focus of this study was to develop a method in order to determine the wear resistance of restorative materials with in-vivo related parameters. Future studies are planned to increase the sample number and to extend this method to other restorative materials.

Clinical results show the potential of conventional GICs as a Class I restorative material [49], but due to the minor mechanical response when subjected to high occlusal loads compared to resin-modified GICs or composites [32], conventional GICs are only indicated for limited load-bearing Class I restorations or as a temporary solution. However, the results presented in this study show that the reinforcement of conventional GICs can improve the mechanical properties in a way that they can withstand higher loads and have the potential to expand their indications to possible permanent restorative solutions. This is particularly relevant for the focus patient group of GICs including children and elderlies, who benefit from the high fluoride release of these materials and the low treatment effort.

The significantly increased wear resistance of DeltaFil may be caused by the dispersed polymeric micelles, which are claimed to prevent crack propagation compared to other conventional glass ionomer restoratives, such as Fuji IX GP and Ketac Universal used in this study. The elastomeric particles can contribute to the fracture toughness of the material by deforming plastically as a result of the increased stresses in front of the crack tip, thus absorbing energy through plastic deformation [50]. This can lead to a blunting of the crack tip and a reduction in the local stress concentration, resulting in the slightly improved fracture toughness and the increased wear resistance observed in this study. This is supported by the fact that no chipping of the restorative material or signs of embrittlement could be observed at the interface between DeltaFil and dentin after termination of the chewing simulation. In this case, no microleakage was observed between the restorative material and the dentin, highlighting the advantage of conventional glass ionomer restorative materials over a composite material for bonding adhesively to the tooth structure without application of an additional bonding system [9]. However, the application of DeltaFil and Fuji IX GP involved an additional step of pre-treatment of the cavity using a conditioner provided by the respective manufacturer that is composed of water and polyacrylic acid. Restorations made by using Fuji IX GP and Ketac Universal resulted in an increased wear rate, indicating the lower fracture toughness compared to DeltaFil. The occurrence of cracks and brittleness at the interface between the restorative material and dentin confirms this.

Wear resistance of Fuji IX GP with additional application of a varnish was not improved compared to the tested materials without application of a protective layer. This is consistent with the results of Brzovic–Rajic et al., who found that the compressive strength after thermal cycling and cyclic loading was not significantly improved by an additional coating [33]. A reasonable high wear resistance of the GIC material itself is a prerequisite to use it without an additional coating for dental restorations to benefit from the advantages of an increased fluoride release without using a protective layer [51].

Future studies should include the comparison of different methods to determine the wear resistance of direct restorative materials in order to enhance the validity of the results that were obtained during this study. Furthermore, the test protocol developed in this study could be extended to enable a comparison with other restorative materials such as resin-modified glass ionomer materials or composites. Chewing simulation testing with different cavity classes could also be included in future studies to provide a more comprehensive clinical picture of direct restorative therapy using conventional glass ionomer materials.

## 5. Conclusions

This study shows that laboratory wear simulations are a useful tool to study basic wear mechanisms and to compare different restorative materials in a controlled setting including in-vivo related parameters. Although laboratory testing using a chewing simulator lacks evidence to predict clinical wear, the aim of in-vitro testing should be to roughly assess the clinical wear properties of a dental restorative material prior to insertion into the oral cavity. DeltaFil, a conventional glass ionomer restorative material with dispersed elastomeric micelles, improved the fracture toughness and wear resistance compared to other conventional GICs, indicating the high potential of this glass ionomer material for permanent Class I restoration.

## Figures and Tables

**Table 1:** Material compositions and powder/liquid ratios of the used glass ionomer restorative materials.

Material	Composition	Powder/Liquid
DeltaFil (DMG)	**Powder:** fluoroaluminosilicate glass, polyacrylic acid **Liquid:** polyacrylic acid, tartaric acid, PEG-PU micelles, water	370 mg/75 mg (ratio: 4.9/1.0)
Ketac Universal (3M)	**Powder:** oxide glass **Liquid:** copolymer of acrylic acid—maleic acid, tartaric acid, benzoic acid, water	339 mg/106 mg (ratio: 3.2/1.0)
Fuji IX GP (GC)	**Powder:** fluoroaluminosilicate glass, polyacrylic acid **Liquid:** polyacrylic acid, polybasic carboxylic acid, water	400 mg/110 mg (ratio: 3.6/1.0)

**Table 2:** Abrasion volume and cycle number of the restored teeth at the termination of the chewing simulation.

Material	Probe	Abrasion Volume in mm^3^	Cycle Number
DeltaFil	1	4.34	1,200,000
	2	3.81	1,200,000
	3	8.55	1,200,000
	4	4.02	1,200,000
	5	5.34	1,200,000
	6	4.89	1,200,000
	7	3.77	1,200,000
	8	4.11	1,200,000
	**Median (IQR)**	**4.23 (3.86–5.23)**	**1,200,000 (1,200,000–1,200,000)**
Ketac Universal	1	8.97	668,629
	2	6.89	1,200,000
	3	16.35	902,660
	4	11.11	1,200,000
	5	18.20	1,200,000
	6	13.63	668,629
	7	13.35	565,098
	8	13.25	565,098
	**Median (IQR)**	**13.30 (9.51–15.67)**	**785,645 (590,981–1,200,000)**
Fuji IX GP	1	8.77	1,200,000
	2	15.68	1,200,000
	3	14.52	1,200,000
	4	15.16	518,602
	5	14.79	419,892
	6	14.67	419,892
	7	15.07	518,602
	8	14.01	737,351
	**Median (IQR)**	**14.73 (14.14–15.14)**	**627,977 (444,570–1,200,000)**

**Table 3:** Mean fracture toughness of the investigated GICs measured using a notchless triangular prism testing rig.

Material	Fracture Toughness in MPa∙m^0.5^ (Mean ± SD)
DeltaFil (*n* = 16)	0.333 ± 0.066
Ketac Universal (*n* = 12)	0.278 ± 0.059
Fuji IX GP (*n* = 17)	0.277 ± 0.051

## Data Availability

The data that support the findings of this study are available from the corresponding author upon reasonable request.
